# Leptin in Human Milk and Child Body Mass Index: Results of the Ulm Birth Cohort Studies

**DOI:** 10.3390/nu11081883

**Published:** 2019-08-13

**Authors:** Chad A. Logan, Linda P. Siziba, Wolfgang Koenig, Prudence Carr, Hermann Brenner, Dietrich Rothenbacher, Jon Genuneit

**Affiliations:** 1 Institute of Epidemiology and Medical Biometry, Ulm University, 89079 Ulm, Germany; 2 Center for Pediatric Research Leipzig, Hospital for Children and Adolescents, University of Leipzig Medical Center, 04103 Leipzig, Germany; 3 Department of Internal Medicine II - Cardiology, University Medical Center Ulm, 89081 Ulm, Germany; 4 Deutsches Herzzentrum München, Technical University of Munich, Munich, Germany and DZHK (German Centre for Cardiovascular Research), partner site Munich Heart Alliance, 80636 Munich, Germany; 5 Division of Clinical Epidemiology and Aging Research, German Cancer Research Center (DKFZ), Im Neuenheimer Feld 581, 69120 Heidelberg, Germany

**Keywords:** leptin, human milk, growth

## Abstract

The objective of the study was to investigate the potential association of human milk leptin concentrations with child body mass index (BMI) and BMI trajectory patterns up to two years of age among children in the Ulm SPATZ Health Study. Leptin concentration was measured in skimmed human milk by ELISA (R&D System). Child BMI was determined at two to three days, three to four weeks, four to five months, one year, and two years of age. In SPATZ, leptin concentration at six weeks was inversely associated with child BMI at four to five weeks [beta –0.13, 95%CI –0.21;–0.05)] and at three to four months –0.12 –0.21;–0.03)]. Among infants of average BMI shortly after delivery, six week leptin was positively associated with greater increase in BMI from four to five weeks up to two years of age [0.16 (0.04;0.27)]. No associations were observed for six month leptin. Direction of association was the same in the Ulm Birth Cohort Study (UBCS), but statistically insignificant as the point estimate included the null effect value. Our results from SPATZ suggest human milk leptin may play a role in early infant growth. However, it is plausible that the lack of associations in UBCS suggest that these differences of human milk leptin composition between populations could have an impact in infant growth and development in a given population.

## 1. Introduction

Early infant weight and length (which represent fetal growth) is a known precursor of weight gain or obesity in childhood (which potentially represent catch-up growth) [1] and therefore may have long-lasting implications on cardiometabolic health. Breastfeeding is often the exclusive, or primary, source of nutrition in early infancy and has been suggested as a potential influencer of infant and child growth. Infants whose early diet does not consist predominantly of human milk are more likely to be larger during infancy and overweight later in childhood [2,3,4,5,6]. 

Among breastfed infants, longer duration of breastfeeding has been widely associated with lower risk of being overweight or obese in child and adulthood [7,8]. These effects have been mainly attributed to multiple synergetic mechanisms associated with the composition of human milk [9]. Particularly, leptin, a component in human milk, is highly correlated with maternal adiposity [10,11] and a strong predictor of child overweight status [2]. Furthermore, leptin in human milk likely plays a role in regulation of appetite and food intake during early infancy [12,13,14]. After accounting for maternal body mass index (BMI), several studies have reported an inverse relationship between higher concentration of leptin in human milk and lower growth in early infancy [14,15,16,17,18,19]. However, these studies were either small, consisting of fewer than 30 subjects [14,15,16,18], or relied on a single sample of human milk [17,19]. Nonetheless, we have recently demonstrated that simple adjustment for pre-pregnancy maternal BMI, an essential covariate for significant results in most previous studies, may have biased previous effect estimates [20].

Therefore, our aim was to investigate potential association between leptin concentrations in human milk and child BMI up to two years of age, using data from two large, independent, but methodologically similar birth cohorts. Recruitments were approximately 10 years apart, thus allowing us to replicate analyses across studies. In addition, associations were investigated using two samples of human milk collected at different time points in the more recent cohort.

## 2. Materials and Methods

### 2.1. Study Design and Population

Data were obtained from the Ulm Birth Cohort Study (UBCS) and the Ulm SPATZ Health Study; two methodologically similar birth cohort studies including live newborns and their mothers, recruited from the general population shortly after delivery in the University Medical Center Ulm, Southern Germany, respectively from 11/2000–11/2001 and 04/2012–05/2013. Details can be found elsewhere [21,22]. Exclusion criteria were outpatient delivery, maternal age <18 years, transfer of the newborn or the mother to intensive care immediately after delivery, and/or insufficient knowledge of the German (UBCS and SPATZ), Turkish, or Russian (both in UBCS only) language. At baseline, the UBCS and SPATZ cohorts included 1090 live newborns of 1066 mothers (67% of all 1593 eligible families) and 1006 live newborns of 970 mothers (49% of all 1999 eligible families), respectively. For the purpose of this analysis, the study populations were further restricted to singleton births. Ethical approval was obtained from the ethics board of Ulm University (UBCS: No. 98/2000; SPATZ: No. 311/11) and of the Physicians’ Boards of the states of Baden-Wuerttemberg and Bavaria (both for UBCS only). Participation was voluntary and written informed consent was obtained in each case. Exposure, outcome, and confounder definitions, as well as statistical methods, were identical for both studies unless specifically stated otherwise.

### 2.2. Human Milk Sample Collection and Analysis

Human milk samples were collected from consenting mothers who were actively breastfeeding at the 6 week and 6 month follow-up. Mothers were instructed to manually express or pump (the latter manually or automated) and refrigerate approximately 10 mL of human milk into study-provided collection tubes. This was done at least one hour after the last breastfeeding on the morning (between 9 am and 12 pm, after breakfast and before lunch) of collection from the home by trained study nurses. In rare instances, nurses helped with expression or pumping of human milk. Samples were taken directly to a central laboratory, divided into aliquots, frozen (−80 °C) within 48 hours of collection, and stored until analysis.

### 2.3. Leptin Measurement

Leptin concentrations [µg/l] were measured in 6 week human milk samples in UBCS and in both 6 weeks and 6 months samples in SPATZ. Analysis was performed in the fat-free (skim) phase using commercially available ELISA (R&D Systems). To remove fat, human milk samples were centrifuged for 5 min at 12,350 g in a Heraeus Biofuge (Kendro Laboratory Products GmbH, Langenselbold, Germany). The interassay coefficient of variation (CV) was <7.0% in both studies. All measurements for both studies were conducted in the same lab, by the same technician, and after approximately the same storage duration of the frozen (−80°C) human milk samples (3.5–4.5 years for UBCS, 3–4 years for SPATZ).

### 2.4. Infant/Child Anthropometric Measurements

Infant and child body weight and body length were obtained from examination documentation (“Untersuchungsheft”) recorded during routinely scheduled pediatric appointments at 3 to 10 days, 4 to 5 weeks, 3 to 4 months, 6 to 7 months, 10 to 12 months, and 21 to 24 months of age. Infants were weighed without diapers and children were measured and weighed with minimal clothing (e.g., underwear). 

### 2.5. Potential Covariates and Confounders

Several maternal (age, birth country, parity, education, BMI, history of smoking), birth (gestational age at delivery, delivery mode), and other factors associated with breastfeeding or human milk composition (breastfeeding duration, duration of lactation up to sampling, exclusive or partial breastfeeding at the time of sampling, estimated feedings per day, breastfeeding method (breast or pump), collection time on the day, and time from last breastfeeding) were considered for inclusion in adjusted models for association with child BMI outcomes. All data were obtained from maternal questionnaire at recruitment, hospital records, or, in the case of human milk sample related data, through in person interview at the time of human milk collection. Data on breastfeeding method, time of collection, and time from last feeding were only available in SPATZ. Concurrent BMI was not available in UBCS. Therefore, pre-pregnancy BMI was used as a proxy. 

### 2.6. Statistical Analyses

Chi-square and Kruskal–Wallis tests were performed to identify differences (*p* < 0.05) in demographic and lifestyle characteristics and 6 week leptin concentrations across study cohorts. To assess the impact of missing data and restrictions, characteristics (proportions or means) of each study sample were compared to 95% confidence intervals of the characteristics within the corresponding full cohort. 

Log transformation was used to normalize leptin values for analysis. Nonlinear association between maternal BMI concurrent to human milk sampling and leptin concentration in SPATZ has been previously described in detail [20]. To account for this, as well as associations with breastfeeding frequency and breastmilk fat content, z-scores controlling for these factors were calculated using spline regression (knots at BMI = 20, 25, and 35) for each sampling period [20].

BMI was calculated from the anthropometric measurements using the standard formula (kg/m^2^). BMI results were then converted to z-scores based on the underlying population using separate linear models for each measurement period and adjusted for precise age at measure (days) and child’s sex. Change in BMI was calculated as proportions (BMI_t2_/ BMI_t1_) which were then converted to z-scores, adjusting for child’s gender and the duration (days) between measurements. To ensure associations were reflective of those in the source population, all z-scores were derived relative to their underlying study populations (UBCS or SPATZ) rather than from standardized growth charts [23].

A cross-sectional model was used to investigate the associations between the 6 weeks standardized leptin concentration with child BMI. General linear models were used to investigate associations of 6 week (UBCS and SPATZ) and 6 month (SPATZ only) maternal BMI-standardized leptin z-scores with age- and gender-standardized child BMI scores and consecutive changes in child’s BMI, adjusting for potential confounders or covariates. Factors associated with leptin concentration within any time period, or which were associated with a change (>1 standard error) in the 6 week leptin z-score beta coefficient (in single adjusted models with any growth outcome), were included in final longitudinal adjusted models. 

In SPATZ, separate sensitivity analyses were conducted, restricted to children who were exclusively breastfed up to at least the 3 to 4 week assessment for 6 week leptin or up to at least the 3 to 4 month assessment for 6 month leptin and to children within 1 standard deviation (SD) of mean BMI as measured between 2 and 3 days of age. 

The Benjamini–Hochberg procedure, controlling for the false discovery rate (FDR) [24,25], was used to adjust *p*-values to account for multiple testing using R version 3.5.0 (R Core Team, 2018). Functional boxplots were created using the R functional data analysis (fda) package [26,27]. All other statistical analyses were performed using SAS 9.4 (SAS Institute, Cary, NC, USA). 

## 3. Results

Compared to UBCS, SPATZ mothers (the more recent study) were older, of higher BMI, had more years of formal education, and were less likely to have had a recent history of smoking (Table 1). Full details on the number of participants and the number of infants that were followed up longitudinally and included in each analysis are available in Table 2. In both UBCS and SPATZ, mothers who provided human milk samples at six weeks were higher educated, less likely to have had a history of smoking and less likely to have been overweight or obese compared to their respective full cohort populations [28]. These associations were stronger among SPATZ mothers who provided a human milk sample at six months.

**Figure 1:**
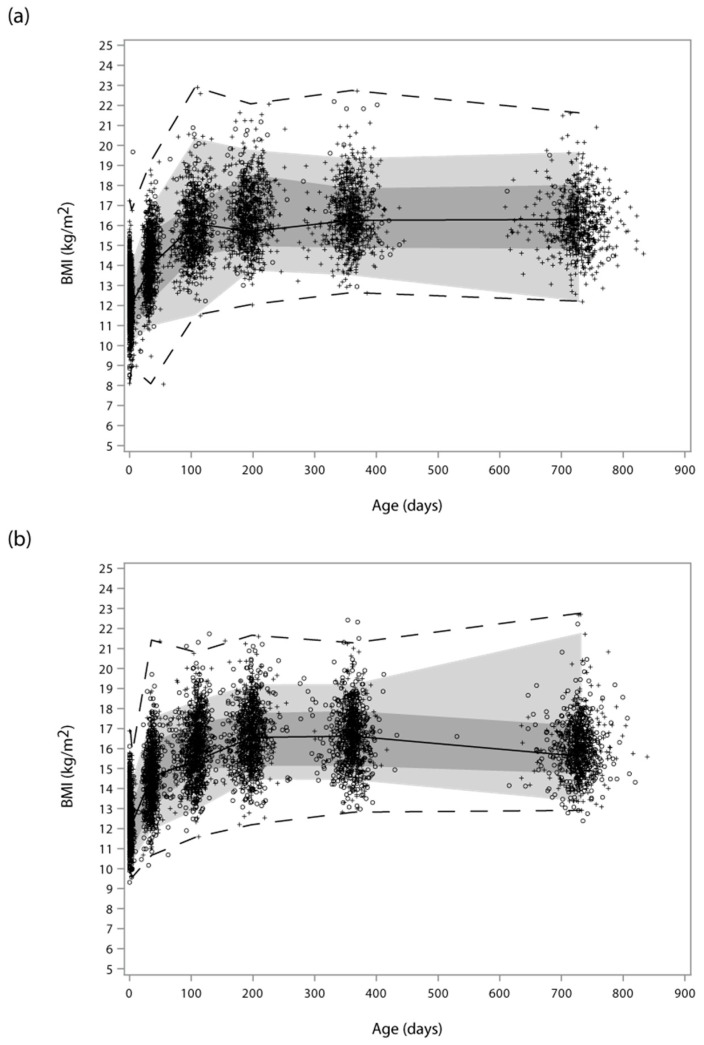
Functional boxplot of child body mass index (BMI) across ages at measure overlaid with scatter plots at each age in the SPATZ (**a**) and UBCS (**b**) cohorts. Scatter plots represent children with complete (+) BMI data used to calculate the functional boxplot and children with incomplete (O) data at one or more measurement periods. (Measurement periods were each collapsed to the average measurement period time for functional boxplot calculation, which is why the functional boxplots exhibit kinks at the center age of each scatter and end at the center age (approximately 750 days) of the last measurement. The solid black line represents the median curve of the functional boxplot. The dark and light grey bands are the central 10% and 50% regions, respectively; the latter corresponding to the interquartile range in a usual boxplot. The dashed lines indicate the nonoutlying envelopes determined by the 1.5 times the 50% central region rule (like the whiskers in a usual boxplot), which in both images correspond to the minimum and maximum since no outliers were detected.).

Median leptin concentrations in six week samples were significantly higher in SPATZ [Median (IQR) = 266.5 (152.0; 498.0) µg/l] than in UBCS [175.0 (79.8; 350.0) µg/l] (*p* < 0.001) (Supplementary Figures S1 and S2). This study effect remained significant after controlling for pre-pregnancy BMI, feedings per day, and demographic differences between cohorts. Singleton infant and child growth measures and patterns were similar in both cohorts (Figure 1). Diminishing increases in BMI were observed up to the six to seven month measurement period, which afterward remained comparatively stable up to the two year measurement in both studies.

In crude and adjusted models, six week standardized leptin concentration in human milk among SPATZ mothers was inversely associated with child BMI up to the three to four month measurement period (Table 3). In the UBCS adjusted models, a marginally statistically significant (*p* < 0.10) inverse association was observed for BMI assessed at four to five weeks with leptin concentration in human milk. In contrast, positive beta coefficients were observed for association of six week leptin with change in BMI from the week four to five measure up to two years in both studies. These were only statistically significant (*p* < 0.05) in SPATZ, following restriction to children whose two to three day BMI was within one standard deviation of the underlying mean BMI. No significant associations were observed for six month leptin concentration. All but one association remained significant following FDR adjustment for multiple comparisons (Table 3).

## 4. Discussion

To our knowledge, this is the largest study to date investigating the relationship between leptin in human milk and early child growth; and also the first study to adjust for nonlinear association of maternal BMI with leptin using maternal weight concurrent to the time of human milk sampling. In SPATZ, higher leptin concentration in human milk collected six weeks postpartum was associated with lower child BMI up to approximately five months of life. We also observed some evidence that higher leptin may be associated with accelerated increases in child BMI during the first two years of life. No statistically significant associations were observed for six month leptin concentration. Though direction of association was the same for all corresponding models in the UBCS cohort, point estimates were weaker and nonsignificant in all cases. 

Our results were similar to findings of several previously published studies, which adjusted for linear pre-pregnancy BMI and also reported negative cross-sectional associations with early infant BMI [14,15,17,18,19]. In sensitivity analyses using this modelling approach, we observed similar but stronger point estimates in SPATZ analyses likely resulting from inaccurate adjustment among mothers with a higher BMI. These results suggest that nonlinear adjustment for maternal BMI used in the current study was more conservative and potentially more accurately accounted for confounding by maternal adiposity. 

Despite these significant findings, it is important to note that at least one recent study which was limited to less than 150 mothers of normal pre-pregnancy BMI [29,30] reported no direct association for six week leptin concentration in human milk with child anthropometric measurements (BMI, skin fold thickness, and body fat percentage) of up to two years. An inverse association similar to that observed in our study was reported for association of leptin measured at four months postpartum with concurrent child weight and lean body mass, but not with these measures at later time periods of up to five years of age. Mostly null findings in these analyses may have been due to the use of crude anthropometrics in statistical models in contrast to the relative measures (for example, z-scores) used in our models. 

However, based on these results and non-replication of our significant SPATZ results in the UBCS cohort, we cannot rule out the possibility that associations may differ by study population or potentially other factors which include, but are not limited to, additional components in human milk. For instance, other appetite hormones such as adiponectin and ghrelin are also related to infant growth patterns [31]. In addition, there is no clearly defined recommendation for an adequate supply of leptin to ensure the most benefit to infants. Therefore, it is possible that the differences observed between these two study populations could impact infant development and growth as well as the infant’s susceptibility to obesity later in life.

Furthermore, we may also be the first to report a possible positive association between leptin in human milk and longitudinal BMI trajectory. These results are in contrast with two smaller studies [14,15], each consisting of less than 30 subjects, which reported negative correlation between leptin concentrations in human milk in the first month of lactation and infant weight gain up to two years. Unlike in these studies, we restricted models to children within one standard deviation of mean early infant BMI. Therefore, children who were more likely to have exhibited extreme (lower or greater) growth velocity due to low or high birth weight were removed. Previous studies also reported only unadjusted correlations, which did not account for several important factors, most prominently maternal BMI. 

Our findings further suggest that leptin concentration in human milk during early lactation is a potential influencer of early infant body mass index, which may also be associated with body composition related growth patterns up to two years of age. Leptin is likely protected from becoming denatured during digestion by favorably high pH in the infant’s digestive tract [12] and/or possibly by binding with soluble leptin receptors [12,32] that are also present in human milk. The ability of leptin to promote satiety has been widely reported in animal experiments [33]. In very early life, exogenous leptin from human milk may promote satiety by supplementing low levels of circulating leptin, which only begin to increase with increasing infant adiposity [14,34]. Further, it has also been suggested that leptin may supplement or even influence hypothalamic activity during a critical period of early development [13,14].

Several limitations should be considered when interpreting our results. First, concentrations of human milk components may differ between mothers, throughout the course of lactation, and can be subject to daily variation [35]. Although we observed little variation between six week and six month leptin concentrations after controlling for several potentially influential factors in the SPATZ cohort, our analyses relied on a single human milk sample from each mother within each sampling period. In addition, although leptin concentrations have been shown to be higher in whole milk than in skimmed milk [36,37], leptin had already been measured in skimmed milk in the UBCS study prior to this research. Thus, to allow replicability between the two cohort studies, leptin was measured in skimmed milk in the SPATZ study. However, it is plausible that the use of skimmed milk in this study could have led to underestimations of leptin concentrations. Nonetheless, besides being synthesized in the mammary gland, leptin is also transferred from maternal plasma circulation to human milk. Thus, granted that both mechanisms may be responsible for the presence of leptin in milk [38], the relative contribution of each mechanism (mammary leptin synthesis and circulating maternal plasma leptin levels) to the final amount of leptin in human milk remains unknown [39]. Moreover, no correlations have been found between absolute leptin concentrations in skimmed milk and whole milk [37]. Therefore, it is unlikely that our results may have been different if whole milk was used for leptin measurements.

Finally, maternal BMI measurements concurrent to human milk sampling were not available in the UBCS cohort. Though we did adjust for pre-pregnancy maternal BMI, it remains unclear whether adjusting for concurrent maternal BMI in UBCS would have better replicated SPATZ results. Although BMI is often used to indirectly estimate adiposity, it is also possible (though not always practical) to directly measure adiposity using other anthropometric measures and/or imaging technology. It is unclear whether results of our study may have differed by using more precise adjustment for maternal adiposity.

## 5. Conclusions

Subject to more complete replication, our results suggest that leptin in human milk may play a role in early infant growth and possibly with changes in BMI in the first year of life. However, further studies are needed to elucidate the potential biological mechanisms for these associations. The differences observed between the two study cohorts warrant further research in leptin concentrations in human milk between populations. Future studies should aim to investigate the significance of synergistic mechanisms, by which additional human milk components such as adiponectin, ghrelin, insulin, and fatty acids may also contribute to, or modify, the associations we report. Finally, more research is needed to understand whether associations observed in our study may have additional long-term implications on child health.

## Figures and Tables

**Table 1:** Demographic characteristics of mothers of singleton births and their children in the Ulm Birth Cohort Study (UBCS) (baseline recruitment 2000–2001) and SPATZ (baseline recruitment 2012–2013) cohorts.

Characteristic	UBCS (*n* = 1042)		SPATZ (*n* = 934)		
	*n*	column%	*n*	column%	*p*-value *
Child’s sex					0.320
Boys	528	50.7%	494	52.9%	
Girls	514	49.3%	440	47.1%	
Delivery mode					<0.001
Vaginal (spontaneous or assisted)	875	84.0%	697	74.7%	
Cesarean (elective or emergency)	167	16.0%	236	25.3%	
Gestation age (weeks) ^†^	1036	40.0 (39.0; 40.0)	933	39.0 (38.0; 40.0)	<0.001
Maternal birth country					<0.001
Germany	828	79.5%	788	85.3%	
Other	214	20.5%	136	14.7%	
Parity (n births of fetus >= 24 weeks)					0.170
0 births	519	50.1%	497	53.3%	
>= 1 birth	516	49.9%	436	46.7%	
Maternal education					<0.001
>= 12 years education	380	37.5%	545	59.6%	
< 12 years education	634	62.5%	370	40.4%	
Maternal age category [years]					<0.001
<= 25	166	15.9%	61	6.5%	
26–35	689	66.2%	643	68.8%	
>= 36	186	17.9%	230	24.6%	
Maternal pre-pregnancy BMI [kg/m^2^]					<0.010
Underweight (BMI < 18.5)	33	3.2%	21	2.3%	
Normal (18.5 <= BMI < 25.0)	696	67.4%	553	61.2%	
Overweight (25.0 <= BMI < 30.0)	218	21.1%	209	23.1%	
Obese (BMI > 30.0)	86	8.3%	120	13.3%	
History of smoking					0.020
No	704	67.6%	670	72.6%	
Yes	337	32.4%	253	27.4%	
Estimated frequency of feedings per day (6 weeks)					0.020
<=5	179	(21.0%)	136	(16.7%)	
>5	673	(79.0%)	678	(83.3%)	

* *p*-Values are chi-square for association with categorical variables or Kruskal–Wallis for association with continuous variables. Bold indicates significant association (*p* < 0.05). ^†^ Results for gestational age presented as median (25th percentile; 75th percentile). Sums may not add up to total because of missing values for some variables.

**Table 2:** Participation of children in the UBCS and SPATZ analyses.

Category	UBCS (*n*)	SPATZ (*n*)	
Enrolled	1090	1006	
Singletons	1042	934	
Leptin measurement	6 weeks	6 weeks	6 months
Human milk sample provided	756	694	476
Leptin measured	747	668	445
Remaining following leptin standardization *	671	587	378
Child BMI (exclusively breastfed **)			
3–4 weeks	576 (572)	554 (552)	372 (332)
3–4 months	590 (573)	555 (549)	375 (336)
6–7 months	589 (565)	531 (523)	359 (336)
1 year	555 (527)	519 (511)	353 (328)
2 years	535 (503)	485 (477)	335 (312)

* Standardization model required complete data on child’s age at sampling, maternal BMI, and estimated feedings per day; ** exclusive up to 3–4 week period for 6 week leptin and up to 3–4 month period for 6 month leptin.

**Table 3:** Adjusted* beta coefficients and 95% confidence intervals for the association between 6 week human milk leptin concentration z-score and child BMI or change in BMI z-scores up to age 2 years.

Parameter	Standardized Human Milk Leptin Concentrations			
	UBCS	SPATZ		
	All Children β (95% CI)	All Children β (95% CI)	Exclusively Breastfed at 3–4 Weeks β (95% CI)	2–3 Day BMI z-score (–1 to +1) β (95% CI)
*Cross-sectional models (BMI measurement)*				
Week 4 to 5	–0.07 (–0.14; 0.01)	-0.13 (–0.21; –0.05)^β†^	–0.17 (–0.26; –0.08) ^β†^	–0.17 (–0.27; -0.07)^β†^
Month 3 to 4	–0.03 (–0.11; 0.05)	-0.12 (-–0.21; –0.03)^β†^	–0.16 (–0.27; –0.06)^β†^	–0.13 (–0.24; -0.02)^α†^
Month 6 to 7	–0.01 (–0.08; 0.07)	-0.05 (–0.14; 0.04)	–0.09 (–0.19; 0.01)	–0.01 (–0.12; 0.10)
*Longitudinal models (change in BMI from the week 4 to 5 measure)*				
Up to month 3 to 4	0.04 (–0.05; 0.13)	0.04 (–0.04; 0.13)	0.08 (–0.02; 0.18)	0.02 (–0.08; 0.12)
Up to month 6 to 7	0.06 (–0.02; 0.13)	0.07 (–0.01; 0.15)	0.06 (–0.03; 0.16)	0.14 ( 0.04; 0.24)^β†^
Up to 1 year	0.02 (–0.06; 0.10)	0.03 (–0.06; 0.11)	0.02 (–0.07; 0.12)	0.10 ( 0.01; 0.20)^α^
Up to 2 years	0.05 (–0.04; 0.13)	0.09 ( 0.00; 0.19)	0.09 (–0.02; 0.20)	0.16 ( 0.04; 0.27)^β†^

* BMI measure z-scores were adjusted for sex and age at sampling or between sampling periods (longitudinal models). Values shown for UBCS are pre-pregnancy BMI-standardized and for SPATZ are maternal BMI-standardized leptin z-scores. All models were additionally adjusted for maternal birth country, education, age at delivery, and history of smoking in the year before pregnancy. All models except those restricted to 2–3 day BMI z-score –1 to +1 were additionally adjusted for child BMI at delivery (UBCS) or at 2–3 days of age (SPATZ). α *p*-value < 0.05, β *p*-value < 0.01, † remains statistically significant (*p* < 0.05) following false discovery rate (FDR) adjustment.

## Supplementary Materials

The following are available online at https://www.mdpi.com/2072-6643/11/8/1883/s1, Figure S1: Distribution of leptin concentrations (µg/l) in human milk samples collected at six weeks in the UBCS and SPATZ cohort studies., Figure S2: Distribution of non-standardized log-transformed and standardized leptin concentrations (µg/l) in human milk samples collected at six weeks in the UBCS and SPATZ cohort studies.
