# Supplementary material for: Leptin in Human Milk and Child Body Mass Index: Results of the Ulm Birth Cohort Studies
Source: Nutrients. 2019 Aug 13;11(8):1883. doi: 10.3390/nu11081883 (PMC6723424; doi:10.3390/nu11081883)
Supplement: Supplementary file 1 [file nutrients-11-01883-s001.pdf]

nutrients

Supplementary figures

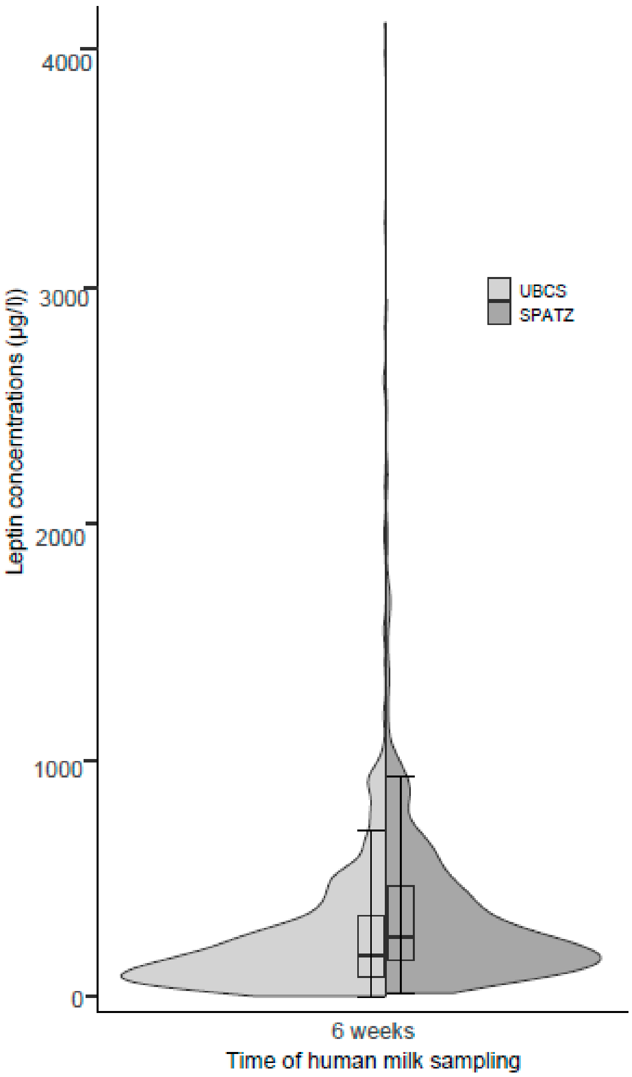

Figure 1: Distribution of leptin concentrations (µg/l) in human milk samples collected at 6 weeks in the UBCS and SPATZ cohort studies. Shaded areas show split violin plots of the density function with inserted boxes indicating the 1st and 3rd quartile and the median.

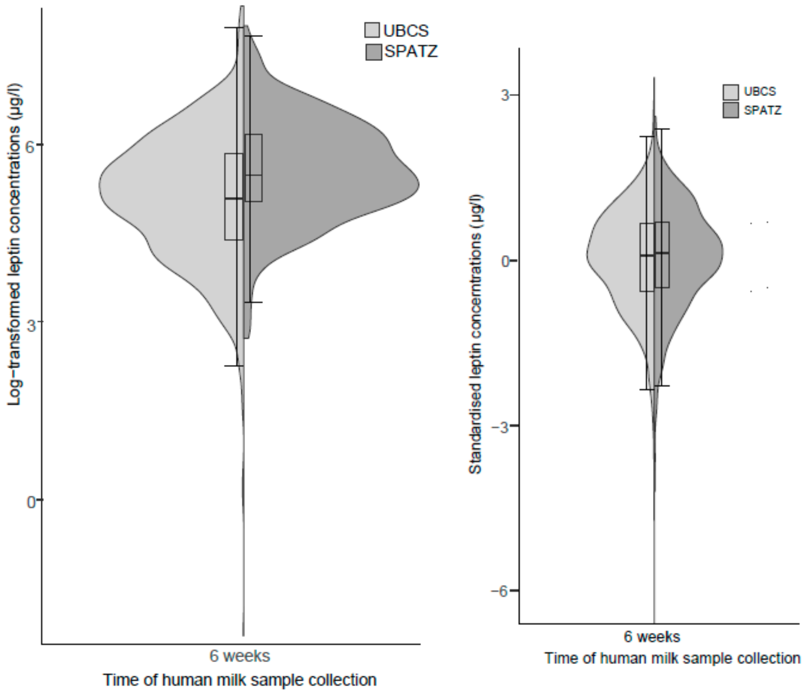

Figure 2: Distribution of non-standardised log-transformed and standardised leptin concentrations (µg/l) in human milk samples collected at 6 weeks in the UBCS and SPATZ cohort studies. Shaded areas show split violin plots of the density function with inserted boxes indicating the 1st and 3rd quartile and the median. Values shown for UBCS are pre-pregnancy BMI standardised and for SPATZ are maternal-BMI-standardized leptin z-scores.
